# Supplementary material for: Neuronal Grin2c expression and the cocaine engram: a medial prefrontal cortex substrate for conditioned place preference
Source: bioRxiv. 2026 Sep 8:2026.09.03.749214. Preprint. [Version 1] doi: 10.64898/2026.09.03.749214 (PMC13622513; doi:10.64898/2026.09.03.749214)

Figure S1

A. Cocaine CPP prior to FACS sorting and qPCR

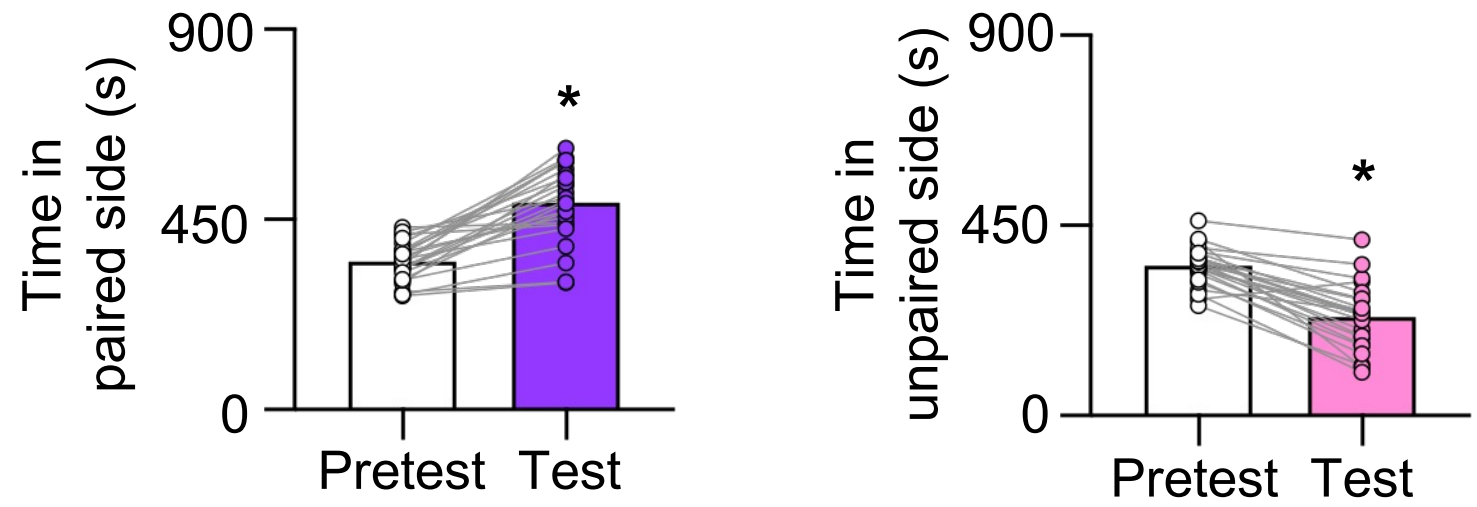

Figure S2

A. Cocaine CPP prior to RNAScope *in situ* hybridization

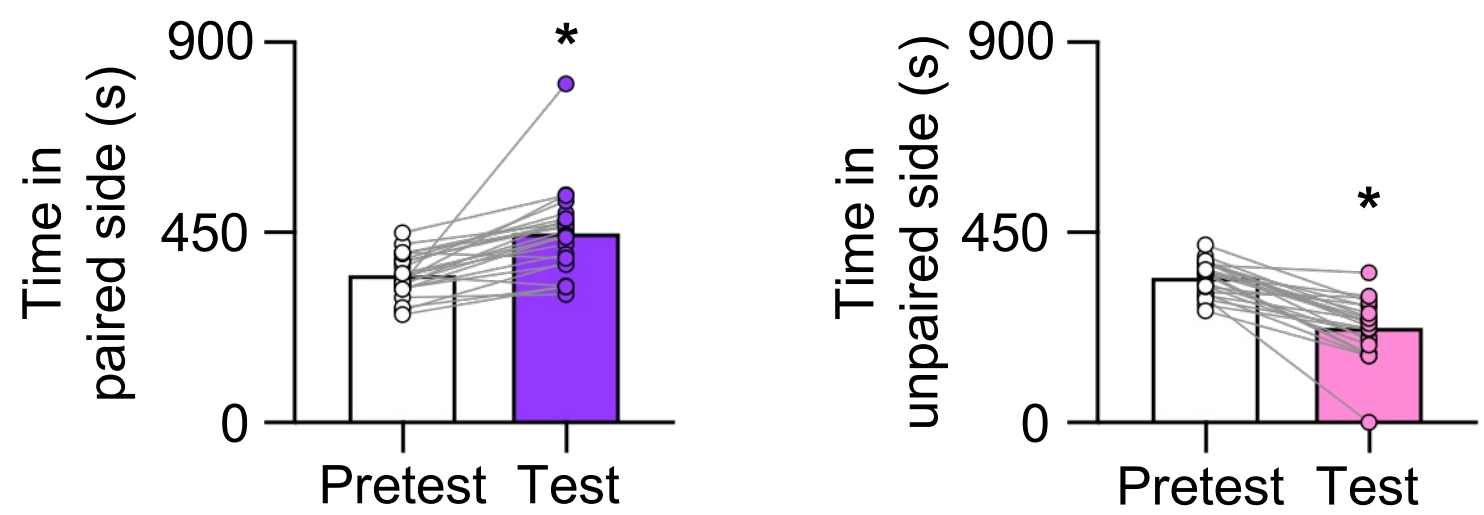

Supplement: Supplementary file 1 [file NIHPP2026.09.03.749214V1-supplement-1.pdf]
